# Mapping the Genetic Landscape of Psychiatric Disorders With the MiXeR Toolset

**DOI:** 10.1016/j.biopsych.2025.02.886

**Published:** 2025-02-19

**Authors:** Dennis van der Meer, Guy Hindley, Alexey A. Shadrin, Olav B. Smeland, Nadine Parker, Anders M. Dale, Oleksandr Frei, Ole A. Andreassen

**Affiliations:** Centre for Precision Psychiatry, Division of Mental Health and Addiction, Oslo University Hospital and Institute of Clinical Medicine, University of Oslo, Oslo, Norway (DvdM, GH, AAS, OBS, NP, OF, OAA); Psychosis Studies, Institute of Psychiatry, Psychology and Neurosciences, King’s College London, London, United Kingdom (GH); KG Jebsen Centre for Neurodevelopmental Disorders, University of Oslo, Oslo, Norway (AAS, OAA); Department of Radiology, School of Medicine, University of California, San Diego, La Jolla, California (AMD); Department of Psychiatry, University of California, San Diego, La Jolla, California (AMD); Department of Neurosciences, University of California, San Diego, La Jolla, California (AMD); Department of Cognitive Science, University of California, San Diego, La Jolla, California (AMD); Multimodal Imaging Laboratory, University of California, San Diego, La Jolla, California (AMD); and Center for Bioinformatics, Department of Informatics, University of Oslo, Blindern, Oslo, Norway (OF).

## Abstract

Psychiatric disorders have complex genetic architectures with substantial genetic overlap across conditions, which may partially explain their high levels of comorbidity. This presents significant challenges to research. Genome-wide association studies (GWASs) have uncovered hundreds of loci associated with single disorders, but the genetic landscape of psychiatric disorders has remained largely obscure. Moving beyond the conventional infinitesimal model, uni-, bi-, and trivariate MiXeR tools, applied to GWAS summary statistics, has enabled us to more comprehensively describe the genetic architecture of complex disorders and traits and their overlap. Furthermore, the GSA-MiXeR tool improves biological interpretation of GWAS findings to better elucidate causal mechanisms. Here, we outline the methodology that underlies the MiXeR tools together with instructions for their optimal use. We review results from studies that have investigated the genetic architecture of psychiatric disorders and their overlap using the MiXeR toolset. These studies have revealed generally high polygenicity and low discoverability among psychiatric disorders, particularly in contrast to somatic disorders. There is also pervasive genetic overlap across psychiatric disorders and behavioral traits, while their overlap with somatic traits is smaller, consistent with differences in polygenicity. Finally, GSA-MiXeR has quantified the contribution of gene sets to the heritability of psychiatric disorders, prioritizing small, biologically coherent gene sets. Together, these findings have implications for our understanding of the complex relationships between psychiatric disorders and related traits. MiXeR tools have provided new insights into the genetic architecture of psychiatric disorders, generating a better understanding of their underlying biological mechanisms and potential for clinical utility.

Our understanding of the genetic underpinnings of psychiatric disorders has improved considerably in the past decade (1). Genome-wide association studies (GWASs) have revealed that these disorders are among the most polygenic of complex human traits (see Table 1); the heritability of psychiatric disorders has contributions from thousands of distinct common genetic variants with relatively small effects compared with somatic traits and diseases (1,2). GWAS discoveries to date only represent the tip of the iceberg, with most genetic risk variants still to be discovered (2).

Statistical genetics tools can characterize the genetic architecture of complex traits beyond GWAS discoveries, including the patterns of overlapping genetic associations across multiple traits. This is of particular relevance for psychiatric disorders given their substantial comorbidity, which is an increasing global health care challenge (3,4). These disorders show strong overlap in clinical features and high comorbidity, likely partially explained by shared genetic predisposition (1), which reflects pleiotropy (5). Genomic studies have revealed extensive genetic correlation across psychiatric disorders and related behavioral and somatic traits (5-7). However, the genetic correlation metric is insufficient to fully characterize genetic overlap between 2 complex traits. This metric represents the correlation of genetic effects across the whole genome. It is a summary measure that builds on the infinitesimal model, which assumes that all genetic variants influence a given trait (8,9). It is possible for 2 traits to be influenced by a shared set of genetic variants with a mixture of concordant and discordant effects, resulting in a genetic correlation that may be close to zero despite extensive shared genetic architectures. We need to better characterize this phenomenon of mixed effect directions (10,11) to increase our understanding of the complex genetic relationship between psychiatric disorders and related traits.

In addition to mapping genetic architecture, downstream analyses of GWAS data have enabled biological insights by coupling genetic discoveries to molecular mechanisms that underlie psychiatric disorders (12-15). However, complex features of the genome such as linkage disequilibrium (LD) (see Table 1) impede the accurate translation of GWAS discoveries to causal mechanisms (16,17). Gene-set enrichment analyses (GSEAs) address these challenges by using GWAS summary statistics to identify associations between a phenotype and groups of genes with common functions or expression patterns. However, the inferential framework of prominent GSEA approaches precludes the calculation of meaningful gene-set–level effect sizes. They are reliant on estimates of statistical significance that prioritize large, nonspecific gene sets that are challenging to investigate empirically (18).

Standard GWAS-based analyses have left an incomplete understanding of the genetic landscape of psychiatric disorders, and biological interpretation following GWAS discoveries is incomplete. Fortunately, the increasing availability of genetic data from massive samples is being complemented by the development of sophisticated analytical tools. Among this new wave of statistical genetics approaches, the MiXeR toolset has contributed to a more nuanced understanding of the genetic architecture of psychiatric disorders (19). Specifically, this toolset enables researchers to characterize the global genetic architecture of complex traits beyond heritability through univariate MiXeR (19) and examine genetic overlap between traits beyond genetic correlations through bivariate and trivariate MiXeR (10,20). Furthermore, MiXeR has improved biological interpretation from GWAS summary-level data through GSA-MiXeR, which identifies putative causal molecular mechanisms (18).

In this review, we aim to provide a conceptual introduction to the MiXeR toolset together with a practical guide to its application before reviewing key insights into psychiatric disorders’ genetic architecture and underlying biology. We will further discuss the main implications of MiXeR findings and the need for additional developments.

## TOOLS OF THE TRADE

### From Infinitesimal Statistics to Causal Mixture Modeling

When analyzing genome-wide data, the most common approach assumes that all genetic variants have some effect on a given trait, with effect sizes forming a single normal distribution. This distribution therefore encompasses infinitesimally small genetic effects and is thus referred to as the infinitesimal model. This elemental model, first proposed by Ronald Fisher in the early 1900s (21), is the basis of a range of statistical genetics approaches that have been developed during the last hundred years, including the widely used LD score regression (LDSC) tool for the estimation of single nucleotide polymorphism (SNP)–based heritability (univariate measure), genetic correlation (*r*_g_; bivariate measure), and enrichment analyses, which measure heritability within predefined subsets of variants (stratified LDSC) (6,22,23).

**Figure 1.**
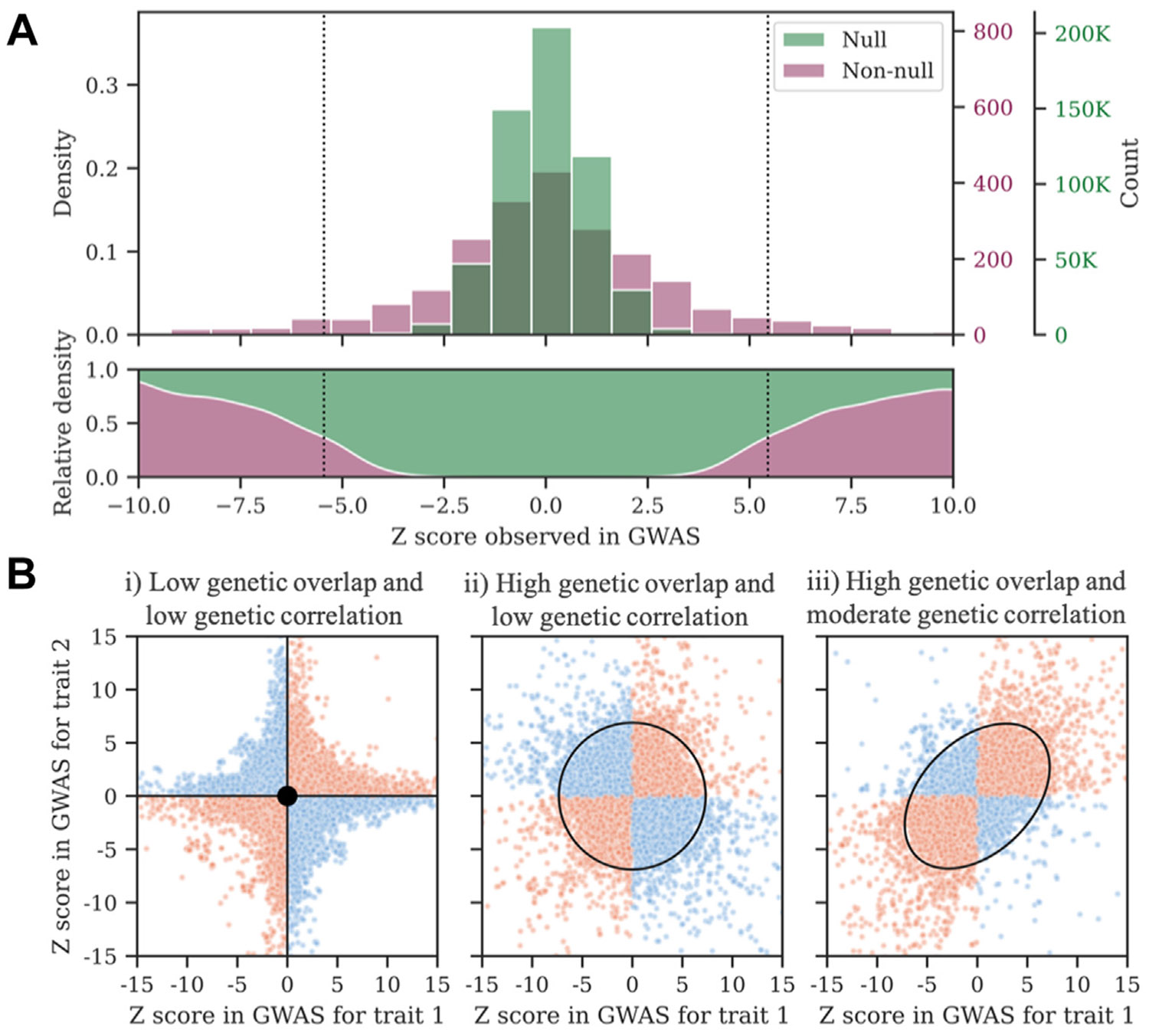
Conceptual understanding of the MiXeR approach. The key concept underlying the MiXeR approach is that, for a given trait, genetic variants can be categorized into either null or non-null rather than assuming that all variants have an effect on the trait (as with the infinitesimal model). Univariate MiXeR models the distinct distributions of non-null and null variants for a single phenotype. **(A)** The *z* scores of null variants (green) predominate around 0, and the effects of non-null variants (purple) predominate toward the tails, while these separate distributions are both centered at 0. The vertical dotted lines thereby denote genome-wide significance at *z* = 5.45 (*p* = 5 × 10^−8^). Modeling the non-null distribution from genome-wide association study (GWAS) summary statistics enables the estimation of the number of non-null variants for a given phenotype (polygenicity) and the average magnitude of non-null effect sizes (discoverability). These parameters are reflected in the shape of the non-null distribution. Additionally, the product of a trait’s polygenicity and discoverability is proportional to its single nucleotide polymorphism–based heritability. **(B)** For bivariate MiXeR, MiXeR models the bivariate distribution of null variants in both traits (central dot), non-null variants in trait 1 (horizontal line) and non-null variants in trait 2 (vertical line) as shown in (i), as well as shared non-null variants (circle and oval) as shown in (ii) and (iii). MiXeR is able to identify genetic overlap with balanced mixed effect directions (ii) whereby a balance of concordant (red data points) and nonconcordant (blue points) shared variants result in an absence of genetic correlation but extensive genetic overlap. MiXeR is also able to identify mixed effect directions for 2 positively correlated traits (iii), whereby the discordant shared variants lower the positive correlation between the traits. MiXeR’s approach to handling mixed effect directions thus enables a more comprehensive understanding of the genetic relationship between 2 traits. Details about the simulation of the data that underlie this figure are provided in the Supplement.

The assumption of the infinitesimal model that all genetic effects are drawn from one distribution prohibits the modeling of several important features of a trait’s genetic architecture, such as polygenicity. In contrast, the MiXeR model follows the assumption that genetic variants can be separated into those that have some effect on a given trait and those that do not have any effect (19), allowing for a more informative mapping of the genetic architecture of complex traits (Figure 1). These classes of variants have been termed non-null and null variants (Bayesian terminology) as well as causal and noncausal variants (fine mapping terminology) (10,19,24). By shifting to a paradigm focused on a mixture of null and non-null effects, it becomes possible to quantify several additional components of a phenotype’s genetic architecture, including polygenicity, average magnitude of effect sizes across non-null variants (also known as discoverability), and genetic overlap irrespective of genetic correlation (see Figure 1 and Table 2).

### Mapping Genetic Architecture With MiXeR

MiXeR constructs a null/non-null framework through Gaussian mixture models. These are Bayesian probabilistic models that model a mixture of distributions (25), with model parameters inferred from GWAS summary statistics.

Univariate MiXeR enables quantification of key features of a trait’s global genetic architecture. It works by modeling the genetic effect of a given variant on a trait (β_j_) as a mixture of null and non-null distributions (Figure 1) (19). The Gaussian non-null distribution is defined by the trait’s polygenicity (π) and discoverability (σ^2^β) (see Table 2). These model parameters are estimated using maximum likelihood estimation, which identifies the best-fitting values by comparing model predictions to observed data, i.e., GWAS summary statistics. Once these primary parameters have been estimated, it is possible to derive several additional measures of interest from the univariate model. First, SNP-based heritability on the observed scale (*h*^2^_SNP_) can be estimated from the product of a trait’s polygenicity and discoverability. Second, to aid interpretation, an additional metric of polygenicity is computed, expressed as the number of strongest non-null variants required to explain 90% SNP-based heritability (nc@p9). A threshold of 90% is applied to prevent extrapolating model parameters into variants with vanishingly small effects. Third, it is possible to use univariate MiXeR parameters to extrapolate how the proportion of SNP-based heritability explained by genome-wide significant variants increases with GWAS sample size, allowing the estimation of the sample size required to identify all genetic loci associated with a given trait.

Bivariate and trivariate MiXeR build on the univariate output to enable estimates of the number of shared non-null variants between pairs or triads of traits. Bivariate MiXeR works by assuming that the genetic effect on 2 traits can be described as a mixture of 4 components: 1) shared non-null effects, 2) unique non-null effects for the first trait, 3) unique non-null effects for the second trait, and 4) null effects. Informed by the model parameters from univariate MiXeR, maximum likelihood estimation is used to estimate the polygenicity and correlation of effect sizes for the shared component between traits (see Table 2). Based on these primary parameters, bivariate MiXeR can derive genome-wide genetic correlation (*r*_g_), the Dice coefficient of polygenic overlap, and the fraction of shared variants with concordant effect direction. Trivariate MiXeR extends this approach to 3 traits (20), also modeling joint overlap among all 3 traits.

### Identifying Gene-Set Effect Sizes With GSA-MiXeR

GSA-MiXeR is a tool for single-trait analysis, building on the MiXeR modeling framework to enable gene-set analysis. It stands out from other GSEA approaches by specifically incorporating the heritability explained by individual genes after accounting for LD and other confounders. This means that GSA-MiXeR can quantify gene-set heritability, i.e., the proportion of the total heritability attributed to a given gene set, and fold enrichment, a measure of the ratio of explained heritability of a gene set to that expected based on a simplified baseline model (see Table 2). These measures provide effect sizes for gene-set enrichment, which has been lacking in prominent GSEA tools.

## ASSUMPTIONS AND PRACTICAL CONSIDERATIONS

**Figure 2.**
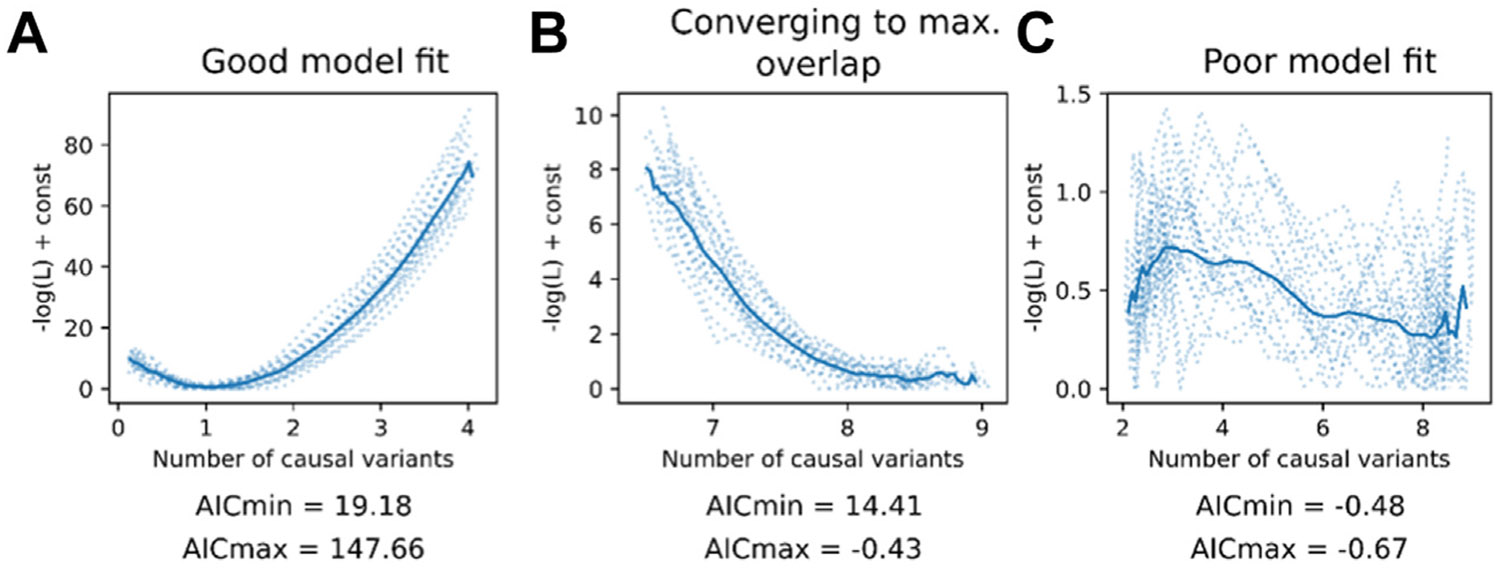
Evaluating bivariate MiXeR model fit using log-likelihood plots. Examples of MiXeR log-likelihood plots, showing negative log likelihood as computed by MiXeR’s likelihood function (y-axis) against the modeled number of shared non-null variants in thousands (x-axis). Each dotted line represents one of the 20 randomized multistart iterations; the solid line indicates the mean curve across all iterations. The lowest point on the mean curve indicates the number of shared non-null variants after parameter optimization. Note that the y-axis scale differs across the 3 plots. From the log-likelihood curves, 2 Akaike information criterion (AIC) values (AICmin, AICmax) are reported, evaluating available evidence for the estimated polygenic overlap vs. alternatives: the minimum and the maximum (max.) possible overlap. **(A)** Example of good model fit, with clear optimum of the log-likelihood function between the minimum and maximum possible overlap, supported by positive AIC values, and similar curves replicated across the iterations. **(B)** Example of good model fit, showing convergence toward maximum possible overlap with stable model fit across the iterations. In this example the AICmin value is positive, indicating that the MiXeR model fits the data better than the minimum overlap model, but AICmax is negative, indicating that the MiXeR modeled overlap is indistinguishable from a scenario of maximum overlap. This pattern is commonly observed in psychiatric disorders and behavioral traits. Larger sample sizes may lead to a clearer divergence between MiXeR-modeled overlap and maximum overlap, although it is possible that the maximum possible overlap represents the true pattern. In the presence of stable model fit across the iterations, it is therefore acceptable to present these findings as long as the implications of the negative AICmax value are discussed (9). **(C)** Example of poor model fit, with no clear optimum, large variation across the iterations, and both AIC values being negative. This implies that one or both phenotypes are either not sufficiently powered for bivariate MiXeR, or their genetic architectures do not sufficiently meet the model assumptions. Therefore, the MiXeR modeled overlap is too unreliable to be reported.

The MiXeR framework is based on several key assumptions. Primarily, MiXeR assumes an additive model of genetic effects with uniform distribution of non-null variants across the genome. Gene-environment interactions, epistasis, and dominance effects are not modeled. Further, uni-, bi-, and trivariate MiXeR assume that non-null variant effect sizes are normally distributed and independent of their allele frequency, LD, and genomic location. These assumptions are likely violated to different degrees for different phenotypes, although sensitivity analyses through simulations outlined in the original work have confirmed that MiXeR is sufficiently robust to the most common violations (10). Nonetheless, model fit should be checked for each analysis, as outlined in Box 1 and Figure 2.

MiXeR tools rely on thorough design of the GWAS producing high-quality summary statistics. Poor representation of some genomic regions, insufficient control for genotyping batches and other confounders, or lack of accurate information about GWAS sample size may distort model results. MiXeR tools are sensitive to the LD structure estimates, which should either come from the same samples as the GWAS summary statistics or from an external reference panel (e.g., 1000 Genomes or Haplotype Reference Consortium [HRC]) that matches the global ancestry of the GWAS.

The additional parameters modeled by MiXeR require well-powered GWASs to ensure reliable results. The statistical power of a GWAS increases with the sample size of the GWAS and the SNP heritability of the trait, but it decreases with the polygenicity of the trait. For bivariate MiXeR, simulations indicate that for highly polygenic traits, a GWAS with an effective sample size of 100,000 combined with an *h*^2^_SNP_ > 0.06 generally generates robust results (11). Lower sample sizes are likely to be sufficient for traits with relatively high discoverability, as is evident from the successful application of uni- and bivariate MiXeR to neuroimaging measures (26).

## APPLYING MiXeR TO REAL DATA

Since the introduction of univariate MiXeR in 2019 (19) followed by the other MiXeR tools in subsequent years (10,20), these tools have been used extensively to study psychiatric disorders, as well as somatic diseases and mental traits (Table S1). Here, we provide guidance on how to interpret key parameters by analyzing and describing some typical use cases before we summarize the literature.

### Univariate MiXeR

#### Interpreting Model Parameters.

**Figure 3.**
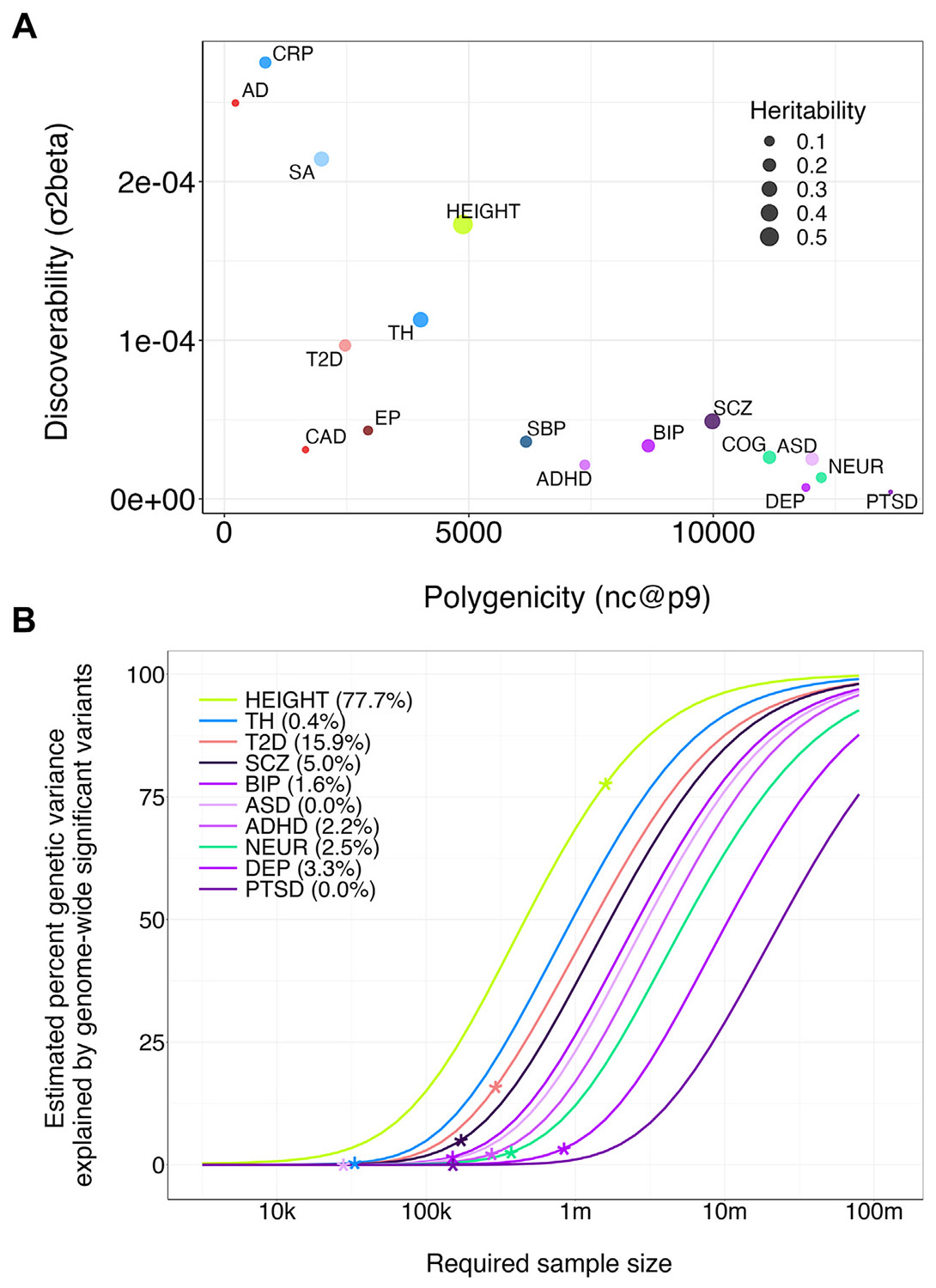
Discoverability, polygenicity, and explained genetic variance, estimated using univariate MiXeR. **(A)** Scatter plot summarizing the discoverability (y-axis), polygenicity (x-axis), and single nucleotide polymorphism (SNP)–based heritability (dot size) of a range of traits that are of relevance for psychiatric genetics. **(B)** A so-called power plot, which leverages the MiXeR parameters to provide estimates of required sample sizes (x-axis) that explain a specific percentage of *h*^2^_SNP_ by genome-wide significant variants (y-axis), as indicated by the colored lines that represent the traits indicated in the legend. The numbers between brackets in the legend indicate the percentage of *h*^2^_SNP_ explained at the current genome-wide association study sample sizes, which correspond to the stars on the lines. Putative biomarkers are colored blue, somatic disorders are red, height is gray, psychological measures are green, and psychiatric disorders are purple. For the sake of clarity, we have only included 1 trait per nonpsychiatric category. AD, Alzheimer’s disease; ADHD, attention-deficit/hyperactivity disorder; ASD, autism spectrum disorder; BIP, bipolar disorder; CAD, coronary artery disease; COG, cognitive performance; CRP, C-reactive protein; DEP, depression; EP, epilepsy; NEUR, neuroticism; PTSD, posttraumatic stress disorder; SA, cortical surface area; SBP, systolic blood pressure; SCZ, schizophrenia; T2D, type 2 diabetes; TH, cortical thickness.

Here, we applied univariate MiXeR to a selection of representative psychiatric, somatic, and psychological traits, plotting each trait’s polygenicity against its discoverability as shown in Figure 3A (GWAS details are provided in Table S2). For example, for schizophrenia, it is estimated that 10,400 genetic variants explain 90% of its *h^2^*_SNP_ (polygenicity, nc@p9) and that the variance of effects for non-null variants is 4.9 × 10^−5^ (discoverability, σ^2^β). This is relatively polygenic, with weak effects per non-null variant, compared with other traits (cf. C-reactive protein [CRP]). The discoverability parameter is also the primary input for the power plots derived from univariate MiXeR (Figure 3B). This demonstrates the relative progress of GWAS for each phenotype and the scale of the studies needed to discover higher proportions of each phenotype’s total SNP-based heritability. For example, only 5% of schizophrenia’s total SNP-based heritability is explained by the latest GWAS.

#### Literature Review.

The literature to date has revealed that polygenicity and discoverability range over approximately 3 orders of magnitude, with an inverse relationship between the two (7), as is also evident from Figure 3A. Univariate MiXeR has uncovered differences in polygenicity across categories of phenotypes ranging from a few dozen for blood markers to nearly 20,000 for posttraumatic stress disorder (PTSD) (19). There are also differences in polygenicity between psychiatric disorders; depression, autism spectrum disorder (ASD), and PTSD are approximately twice as polygenic as attention-deficit/hyperactivity disorder (ADHD). Similarly, discoverability ranges from 1 × 10^−6^ to 1 × 10^−3^, with these same traits at the extremes. Most psychiatric traits are at the early phase of discovery, with sample sizes over 10 million required to explain over 90% *h*^2^_SNP_ by discovered variants. In contrast, some blood markers are approaching saturation with under 1 million participants.

There are converging lines of evidence that support MiXeR estimates of polygenicity. A recent GWAS of height including 5.4 million individuals demonstrated that the entirety of the trait’s SNP-based heritability could be explained by 9683 quasi-independent SNPs discovered at genome-wide significance (27). This is very similar to a polygenicity of 9400 non-null variants estimated by MiXeR based on a GWAS with one-fifth of this sample size (11). The validity of the estimates is further supported by findings from other tools that also quantify polygenicity, including s-LD4M (28) and BayesS (29).

### Bivariate MiXeR

#### Interpreting Model Parameters.

**Figure 4.**
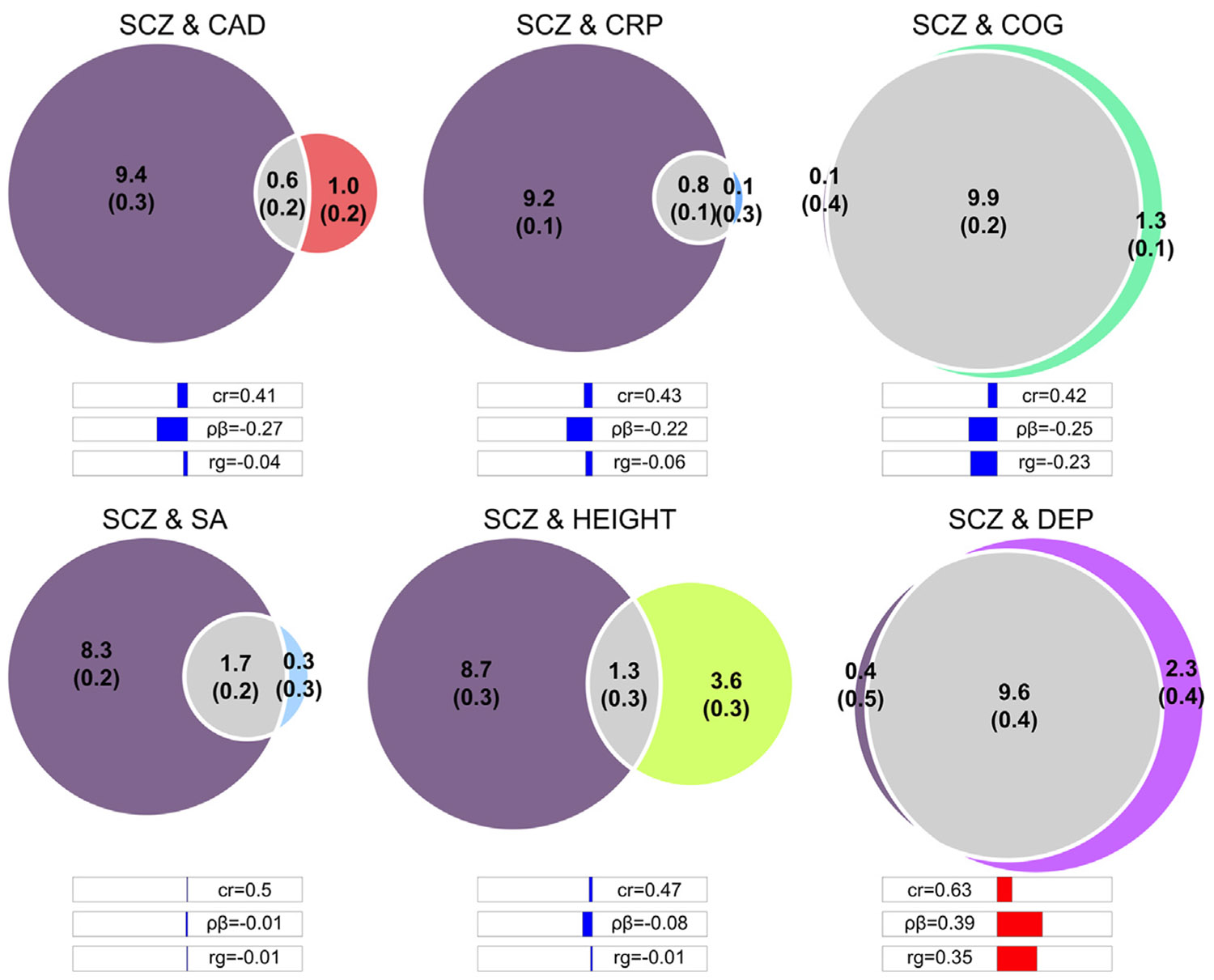
Genetic overlap between schizophrenia (SCZ) and other complex traits. The Venn diagrams illustrate the number of shared non-null variants, in thousands, between SCZ (in purple on the left) and other complex traits, as identified through bivariate MiXeR. The numbers in brackets indicate the estimates’ standard deviation, in thousands, across 20 iterations. Below the diagrams are bars indicating the effect direction concordance rate of shared variants (cr; 0.50 would be a balance between opposite and same direction of effects), the genetic correlation of the shared variants (ρ_β_), and the global genetic correlation (*r*_g_). CAD, coronary artery disease; COG, cognitive performance; CRP, C-reactive protein; DEP, depression; SA, cortical surface area.

We applied bivariate MiXeR to schizophrenia paired with coronary artery disease (CAD), CRP levels, cognition, cortical surface area, height, and depression (Figure 4), which demonstrated several commonly observed patterns of genetic overlap. When interpreting bivariate MiXeR results, the absolute number of shared variants should first be considered in the context of the polygenicity of the 2 traits. This feature can be described numerically by calculating the proportion of each input phenotype’s total polygenicity that is shared. For example, because CAD has a polygenicity of approximately 1600 non-null SNPs, 600 shared variants represent 0.38 of all its non-null variants, indicating that a relatively large proportion of the genetic architecture of CAD is independent of schizophrenia, in contrast to the CRP use case. The Dice coefficient also captures the extent of the overlap across both pairs of traits in a single, weighted metric. While this can be helpful to enable comparisons of overlap across distinct pairs of traits, the Dice coefficient may conceal divergent patterns of overlap if the polygenicity of the input traits varies substantially across pairs. Bivariate MiXeR also provides information about the balance of effect directions among shared variants, which explains why genetic overlap can be observed even in the absence of genome-wide genetic correlation. This is captured both by the proportion of shared variants with concordant effects (cr) and the genetic correlation of the shared variants (ρ_β_), which are presented together with genetic correlation in Figure 4 as 3 bars beneath the Venn diagrams. For example, schizophrenia and depression have nearly complete genetic overlap with 9600 shared variants, while their genetic correlation is 0.35. Given its moderately positive correlation, the concordance rate demonstrates that a small majority of shared variants have the same direction of effects (0.63). The ρ_β_ metric may provide additional insight into the genetic relationship between 2 pairs in scenarios in which there is a greater discrepancy between the number of shared variants and the polygenicity of the 2 traits. For example, for schizophrenia and CRP, the genome-wide correlation is just −0.06, but the correlation of shared variants is −0.22, suggesting that there is a stronger alignment of effects among shared variants between schizophrenia and CRP than is indicated when only evaluating genome-wide genetic correlation.

#### Literature Review.

Bivariate MiXeR has been extensively used to estimate the genetic overlap of psychiatric disorders with other psychiatric disorders (11,13,30,31), substance use disorders (32), cognitive ability (11), sleep phenotypes (33), neurological diseases (34,35), cardiometabolic conditions (36,37), and intermediate biological markers, such as brain magnetic resonance imaging measures (26,38) and blood markers (39,40). This is often done in conjunction with other metrics of genetic overlap, such as genetic correlation and methods to identify specific shared genetic loci (41). For pairs of psychiatric disorders, the proportion of shared variants thereby nearly always indicates considerably greater genetic overlap than that indicated by their genetic correlation. The pattern of overlap between psychiatric disorders and other illnesses seems to differ by disease category, with a larger extent of overlap with neurological conditions than common somatic diseases (35). Moreover, neurological and somatic conditions show less overlap among themselves than that observed among psychiatric disorders (35) and mental traits such as cognitive ability and subjective wellbeing (11), perhaps indicating that this is a characteristic of the most polygenic traits. Most studies combined several tools, providing a more complete overview, and also reported the application of proper quality control steps together with the presentation of several reliability metrics, as recommended.

To the best of our knowledge, there are no other methods directly comparable to bivariate MiXeR. However, there are other methods that recognize the implications of mixed effect directions for estimating genetic overlap. Local analysis of covariance (LAVA) estimates genetic correlation across thousands of genetic loci (42). When applied to psychiatric disorders, these LAVA estimates show high similarity to the effect direction concordance rates derived from bivariate MiXeR across psychiatric disorders (11,43). Genomic structural equation modeling provides an alternative approach to modeling the genetic overlap across multiple traits, although it relies on genome-wide genetic correlations (44). This has primarily been used to identify the underlying latent structure across distinct psychiatric disorders but has also demonstrated significant heterogeneity of genetic effects across disorders, consistent with mixed effect directions (44).

### Trivariate MiXeR

#### Interpreting Model Parameters.

**Figure 5.**
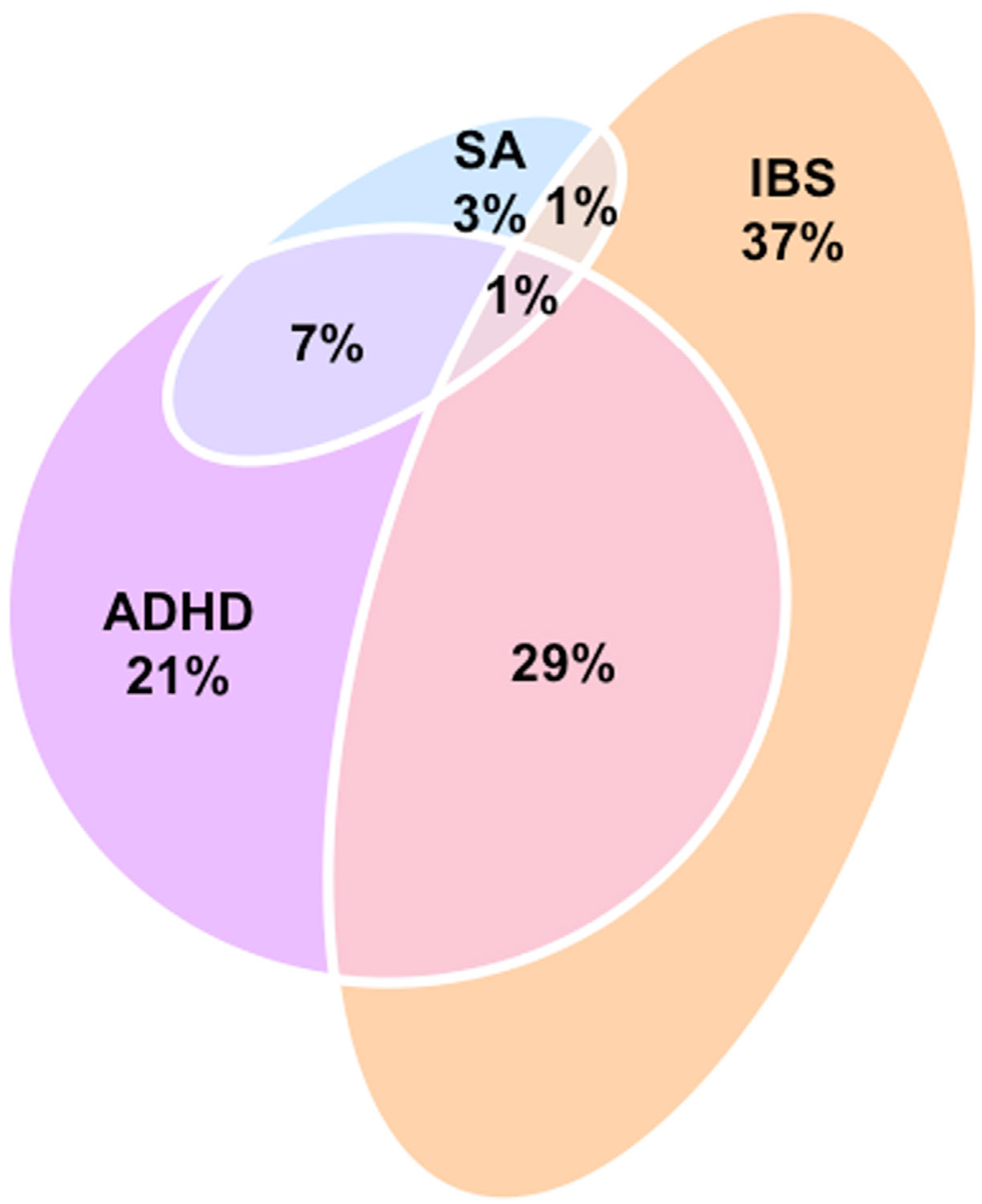
Modeling genetic overlap across 3 complex traits through trivariate MiXeR uncovers nonuniform distributions of bivariate overlap. Euler diagram summarizing the extent of shared and unique genetic components for each of 3 related brain traits, with the numbers listed reflecting the percentage of the total number of causal variants involved across all 3 traits. ADHD, attention-deficit/hyperactivity disorder; IBS, irritable bowel syndrome; SA, cortical surface area.

The model parameters and output metrics of trivariate MiXeR are highly similar to those of uni- and bivariate MiXeR, including the estimates for the individual traits and pairs. Analogous to bivariate MiXeR, the results are most intuitively visualized through Euler diagrams, which capture the extent of shared and unique genetic architectures (Figure 5). Here, the overlap is presented in terms of percentages of the total number of variants across all traits to highlight relative proportions of all areas in the diagram. This shows that the extensive genetic overlap between ADHD and irritable bowel syndrome (sharing 30% of total variants) is almost entirely independent of cortical surface area (which shares 1% of the total), providing insight into the biological relationship between these traits.

#### Literature Review.

Trivariate MiXeR has been used to interrogate the genetic overlap across common patterns of comorbidity between psychiatric, neurological, and somatic conditions (20) and different types of ascertainment and clinical subtypes of bipolar disorder (45). This indicates that bipolar I disorder may have a distinct set of risk variants that distinguish it from the less-severe bipolar II disorder, which is better represented in biobank-based research. Trivariate MiXeR has also been used to identify the extent of overlap across different autoimmune diseases, supporting a polygenic continuum ranging from autoimmune to autoinflammatory conditions (46), thereby informing disease nosology. In addition, trivariate MiXeR findings have indicated that estimates of overlap between 3 traits may be distinct from the overlap deduced from combinations of bivariate estimates, which suggests specific genetic architectures of multimorbidity (20).

As with bivariate MiXeR, we are not aware of any tools directly comparable to trivariate MiXeR. Multivariate tools such as GenomicSEM most closely approach its ability to model shared genetic architecture beyond 2 traits.

### GSA-MiXeR

#### Interpreting Model Parameters.

We illustrate GSA-MiXeR parameters in Table 3, showing the top 5 gene sets for schizophrenia and Alzheimer’s disease based on fold enrichment. This demonstrates how the fold enrichment metric is computed as a ratio of the fraction of heritability derived from the full GSA-MiXeR model (h2_frac) and the heritability from its baseline model (h2_base_frac). This is important because the heritability of a gene set is expected to increase proportionally to its size. For example, “intrinsic component of postsynaptic membrane” captures the highest proportion of schizophrenia’s heritability (0.0295) but comprises 119 genes. Fold enrichment is necessary to identify gene sets that capture disproportionate amounts of heritability given their size and to enable valid intra- and interphenotype comparisons. This is further facilitated by the provided standard errors, which allow for evaluation of the precision of these estimates. This metric reveals that “membrane depolarization during atrioventricular node cell action potential” is the most highly fold-enriched gene set for schizophrenia, despite being the 11th ranked gene set using MAGMA, which only computes statistical significance and lacks a measure of effect size. When compared with Alzheimer’s disease, however, the top schizophrenia gene sets have substantially lower fold enrichments. This suggests that the SNP heritability for Alzheimer’s disease is more concentrated within a smaller number of gene sets than schizophrenia, which may relate to schizophrenia’s greater heterogeneity.

#### Literature Review.

When applied to a range of complex human phenotypes, it has been demonstrated that GSA-MiXeR is able to accurately identify smaller, more biologically relevant gene sets using the fold enrichment metric (47), such as pathways related to dopamine and calcium channel processes and molecular functions, as well as GABAergic (gamma-aminobutyric acidergic) interneuron development in bipolar disorder (45). This is relevant because smaller gene sets may be more feasible to validate experimentally and, ultimately, target pharmacologically; a recent preprint applied GSA-MiXeR to the Drug Gene Interaction Database as part of a wider strategy to prioritize drugs for potential repurposing in psychiatric disorders (48). Because GSA-MiXeR builds on gene-level heritability estimates, users may also define gene sets themselves. This can be leveraged to capture the heritability and fold enrichment of any study-specific particular genomic features of interest, as was recently done for genomic regions that contain pathogenic copy number variants (49).

## DISCUSSION

Moving beyond the infinitesimal model, the mathematical modeling implemented in the MiXeR toolset has enhanced our understanding of the genetic landscape of complex traits (10,18-20). Univariate MiXeR has enabled us to map genetic architectures, moving from general statements about high polygenicity and small effect sizes in psychiatric genetics to quantifying these characteristics for individual traits (19,40). The application of bivariate MiXeR has made clear that the extent of shared variants between complex traits is much larger than that indicated by genetic correlations, which has increased our understanding of the genetics of comorbidity, a major clinical challenge (11,35). Trivariate MiXeR has further improved our ability to investigate the genetic overlap that underlies multimorbidities, providing additional insights into the complex patterns of overlap that underlie these clinically observed relationships (20). Last, GSA-MiXeR has resolved the problems with reliance on statistical significance alone for GSEA by the addition of effect size estimates (18,45), enabling the identification of more specific genetic pathways important for understanding the molecular mechanisms of psychiatric disorders.

By quantifying the extensive polygenicity and low discoverability of psychiatric disorders, MiXeR provides an avenue to a better understanding of these disorders. Polygenicity and discoverability may act as markers of a disorder’s heterogeneity and biological complexity (7), as has been explored with ADHD (11). Polygenicity and discoverability estimates could be used to test whether subphenotyping or novel nosological systems reduce the biological complexity and heterogeneity of psychiatric disorders. Results from bi- and trivariate MiXeR have provided several insights into the genetic architecture of psychiatric disorders. For psychiatric disorders and mental traits, most non-null variants for one trait are associated with a second trait, regardless of the genetic correlation between the 2 traits (11). The pattern of overlap is more variable for less polygenic traits, such as neurological disorders and neuroimaging traits, pointing to the presence of overlapping molecular mechanisms for some but not all of these traits (35,38), in contrast to less-related polygenic traits such as height (11). These estimates indicate that most variants associated with a psychiatric disorder are likely to be nonspecific, affecting a wide range of these disorders and related traits (10). However, effect sizes and effect directions do differ to varying degrees across pairs of traits. Thus, the effect size distribution across large numbers of highly pleiotropic variants may contribute to specificity for a specific psychiatric disorder (11,50), which may partially explain their high rates of comorbidity.

GSA-MiXeR and complementary tools are essential to uncover the biological mechanisms that underlie the identified polygenicity of psychiatric traits and their pleiotropy. Other tools that identify specific shared variants contribute to this further, including conditional and conjunctional false discovery rate analysis (41). This approach has also been extended to polygenic prediction. In the pleioPGS tool, reranking of variants according to their conditional association can improve prediction (51). Similarly, MiXeR-Pred sources pleiotropy to improve predictive accuracy (52), while the new MiXeR-TAG will annotate gene sets to the unique and overlapping regions on the MiXeR Venn diagrams, deriving biological insights. Furthermore, despite the growing availability of GWASs of participants of non-European ancestries, their limited power remains a barrier for MiXeR analysis. Nevertheless, we expect that the number and diversity of well-powered GWASs will grow steadily in the coming years, encouraging analyses of non-European ancestries.

## CONCLUSIONS

The findings generated through the MiXeR toolset have provided a deeper, more nuanced understanding of the interrelationships between complex phenotypes. MiXeR and other tools demonstrate that we may leverage such shared genetic signal, turning this complexity into an advantage. By embracing this complexity, the MiXeR tools will continue to contribute to the advancement of the field of psychiatric genetics.

## Supplementary Material

1

2

Supplementary material cited in this article is available online at doi.org/10.1016/j.biopsych.2025.02.886.

## Figures and Tables

**Table 1.** Glossary of Key Terminology

Term	Definition
Genome-Wide Association Study	An observational study testing for the association of common genetic variants, primarily SNPs, with an outcome using a mass-univariate approach
SNP-Based Heritability	The proportion of phenotypic variance captured by the additive effects of measured, common SNPs
Polygenicity	The number of genetic variants with an effect on a given trait. May also refer to the condition of being influenced by many genes, as opposed to one (monogenicity) or several (oligogenicity)
Genetic Locus	A specific region of the genome, defined by its chromosomal location
Linkage Disequilibrium	Nonrandom inheritance of a given pair of alleles, typically due to their physical proximity in the genome
Genetic Correlation	The correlation of variant effect sizes between 2 phenotypes after controlling for linkage disequilibrium
Pleiotropy	When a genetic variant affects 2 or more phenotypes. In statistical genetics, this term often refers to a statistical association of that variant with more than 1 phenotype (7,53).
Infinitesimal Model	A statistical model first proposed by Ronald Fisher in 1918 that is used by most statistical genetics tools and assumes that the effect sizes of all genetic variants for a given trait are derived from a single normal distribution with effect sizes ranging to infinitesimally small.
Gaussian Mixture	A statistical model that assumes that a given statistical distribution, genetic variant effect sizes in the case of MiXeR, can be represented by 2 or more distinct normal distributions.
Null/Non-Null Variants	The concept that genetic variants can be categorically divided into variants with some true effect on a given trait (non-null) and those with no true effect on a trait, beyond statistical noise (null). Also referred to as causal/noncausal and trait-influencing/not trait-influencing.
Genetic Overlap	The degree to which 2 or more traits share non-null variants regardless of the effect direction of the shared variants.

SNP, single nucleotide polymorphism.

**Table 2.** MiXeR Model Parameters and Derived Metrics

	Denotation	Model Use	Definition
Parameter			
Polygenicity	π	Univariate	Proportion of non-null variants
Discoverability	σ^2^_β_	Univariate	Variance of effect sizes of non-null genetic variants
Polygenicity of Shared Component	π_12_	Bivariate	Proportion of variants with non-null effect across the traits, irrespective of effect direction
	π_123_	Trivariate	
Polygenicity of Unique Components	π_1_, π_2_	Bivariate	Proportion of non-null variants that are unique to 1 trait
	π_1_, π_2_, π_3_	Trivariate	
Baseline Heritability of Each Gene	h2_gene_base	GSA	Fraction of *h*^2^_SNP_ attributed by each gene estimated by a baseline model that assumes that genetic effects are evenly spread across the genome
Estimated Heritability of Each Gene	h2_gene	GSA	Fraction of *h*^2^_SNP_ attributed by a given gene in a full model estimating each gene’s contribution to trait’s heritability
Derived Metric			
Polygenicity	nc@p9	Univariate	Number of non-null variants to explain 90% of *h*^2^_SNP_
Genetic Correlation	*r*_g_	Bivariate	Genome-wide genetic correlation
Correlation of Shared Variants	ρ_β_ or *r*_gs_	Bivariate	The correlation of effect sizes among shared non-null variants
Dice Coefficient		Bivariate	Index of similarity, twice the ratio of the shared polygenicity (π_12_) over the sum of univariate polygenicities of the 2 traits
Concordance Rate	Cr	Bivariate	The fraction of shared non-null variants with concordant effect directions
Fold Enrichment of Gene Sets	Enrich	GSA	Ratio of heritability attributed to a given gene set over its baseline heritability. The null value for fold enrichment is 1.0.

GSA, gene-set analysis; SNP, single nucleotide polymorphism.

**Table 3.** Top 5 Gene Sets Sorted by Fold Enrichment as Estimated by GSA-MiXeR for Schizophrenia and Alzheimer’s Disease

GO Gene Set	NGENES	Enrich	h2_frac	h2_base_frac	MAGMA *p*	MAGMA Rank
Schizophrenia						
Membrane depolarization during AV node cell action potential	5	5.30	0.0062	0.0012	3.96 × 10^−7^	11
L type voltage-gated calcium channel complex	11	3.75	0.0074	0.0020	5.96 × 10^−7^	12
High voltage-gated calcium channel activity	9	3.74	0.0069	0.0018	2.62 × 10^−6^	17
Voltage-gated calcium channel activity involved in cardiac muscle cell action potential	5	3.39	0.0060	0.0018	2.13 × 10^−7^	10
Intrinsic component of postsynaptic membrane	119	1.58	0.0295	0.01860	1.16 × 10^−7^	14
Alzheimer’s Disease						
Negative regulation of amyloid fibril formation	11	34.24	0.0211	0.0006	4.87 × 10^−7^	6
Immune complex clearance	5	20.76	0.0057	0.0003	4.27 × 10^−6^	12
Neurofibrillary tangle	5	17.50	0.0077	0.0004	8.89 × 10^−13^	1
Reverse cholesterol transport	17	14.88	0.0430	0.0029	9.38 × 10^−7^	7
Positive regulation of complement activation	5	10.30	0.0044	0.0004	1.48 × 10^−8^	2

NGENES indicates the number of genes in the gene set; h2_frac indicates the fraction of single nucleotide polymorphism heritability explained by the gene set; h2_base_frac indicates the fraction of single nucleotide polymorphism heritability explained by the gene set using the baseline model; MAGMA *p* indicates MAGMA estimated *p* value; and MAGMA Rank indicates the rank of gene set when sorted on MAGMA *p*.

AV, atrioventricular; GO, Gene Ontology.

## Box 1. How to Apply MiXeR Tools

The following steps broadly make up a successful MiXeR analysis:

1. Select trait(s) of interest—get the latest ancestry-specific genome-wide association study summary statistics.
2. Format summary statistics—standardize column names, ensure inclusion of required columns such as z score and effective sample size, and remove the major histocompatibility complex. This can be achieved through provided convenience functions.
3. Download, install, and run the MiXeR tool of interest—Step-by-step instructions are provided at https://github.com/precimed/mixer, https://github.com/precimed/mix3r, and https://github.com/precimed/gsa-mixer. Using high-performance computing facilities is recommended because MiXeR analyses typically require considerable computational resources, as outlined on the repositories. Precompiled singularity containers are available.
4. Do robustness checks—check Akaike information criterion (AIC) values, the log likelihood, QQ plots, and standard errors:
  - AIC values contrast how well the full MiXeR model fits the observed data compared with a simplified model, controlling for the increased complexity of the full model. A negative AIC value means that the simpler model fits the data better than the MiXeR model, in most cases indicating insufficient statistical power to perform the MiXeR analysis, although there are exceptions (see Figure 2). The Bayesian information criterion can be used in addition to AIC (see the Supplement). The following comparators are used for each model:
    - univariate MiXeR—infinitesimal model;
    - bivariate MiXeR—minimum and maximum possible overlap (constrained by genome-wide genetic correlation and the polygenicity of the least polygenic trait, respectively);
    - trivariate MiXeR—AIC differences for the 3 input univariate analyses and 3 pairwise bivariate analyses underlying each trivariate analysis;
    - GSA-MiXeR = the baseline model (see Table 2).
  - QQ plots: Model fit for univariate and bivariate MiXeR can also be assessed by visually inspecting empirical vs. model-based QQ plots. For univariate MiXeR, this includes standard QQ plots and 3 3 3 stratified minor allele frequency– by linkage disequilibrium–binned QQ plots. Conditional QQ plots are presented for bivariate MiXeR. A large deviation between empirical and model-based curves indicates violation of model assumptions (see the Supplement).
  - Log-likelihood plots and standard deviations of parameter estimates across 20 randomized multistart iterations provide additional ways to evaluate the robustness of MiXeR results. Log-likelihood curves from bivariate MiXeR visualize the stability of the fitted model across multistart iterations, plotting MiXeR’s negative log likelihood against the number of shared non-null variants (see Figure 2 for more details).
  - Standard errors: GSA-MiXeR estimates conventional standard errors for all model parameters to enable evaluation of the precision of its estimates.
