# Supplementary material for: Mapping the Genetic Landscape of Psychiatric Disorders With the MiXeR Toolset
Source: Biol Psychiatry. Author manuscript; Available in PMC 2026 May 20. (PMC13186938; doi:10.1016/j.biopsych.2025.02.886)
Supplement: 1 [file NIHMS2171734-supplement-1.pdf]

SUPPLEMENTARY INFORMATION

Mapping the Genetic Landscape of Psychiatric Disorders With the MiXeR Toolset

van der Meer *et al.*

Simulations

For Figure 1 we generated GWAS summary statistics, using simulated data based on UK Biobank (UKB) imputed genotype data. Simulations were done with the SIMU tool (https://github.com/precimed/simu) using ~50K randomly selected unrelated (up to the second degree of relatedness) individuals and ~780K genotyped variants. For Figure 1a, the univariate MiXeR results, we simulated a trait with h^2_{SNP}=0.4 and π=0.003. We ran GWAS with PLINK2, with 20 principal components included as covariates.

For Figure 1b, the bivariate MiXeR results, we simulated six quantitative traits (two for the non-overlapping scenario, two for overlap without correlation and two for overlap with correlation at r_g=.6). These all had equal heritability (h^2=0.4) and equal polygenicity (π=0.003).

Additional QC procedures

- Bayesian Information Criterion (BIC) is an alternative method for model selection to AIC. BIC is a more conservative measure which has been shown to be overly stringent in scenarios of moderate statistical power . When applied to MiXeR, simulations have shown that AIC differences are a more appropriate test for the evaluating the reliability of MiXeR estimates, showing that the BIC was overly conservative for scenarios of statistical power equivalent to most current MiXeR analysis (1).
- *Conditional QQ plots* demonstrate how well the MiXeR modelled p-values on to observed p-values (Supplementary figure 1). Good model fit is indicated by the modelled enrichment closely mapping on to the observed enrichment. Clear deviation from the observed enrichment may indicate poor model fit and either inflated or underestimated overlap.

Additional considerations when interpreting AIC differences for bivariate MiXeR

In some scenarios, MiXeR may report negative AIC differences for minimum or maximum overlap despite adequate model fit. This is because AIC differences give an indication of the ability to distinguish MiXeR-modelled overlap with a specific comparator, in the case of bivariate MiXeR minimum and maximum overlap. Theoretically, if the true overlap was minimum or maximum overlap, then the AIC difference would therefore be negative but the MiXeR estimate would still be precise. A similar scenario is frequently observed in practice for

psychiatric disorders and related mental traits, in which MiXeR often reports almost complete genetic overlap, alongside a positive AIC difference compared to minimum overlap and a negative AIC difference compared to maximum overlap. Such a scenario can be distinguished from poor model fit by visualising the log-likelihood plots, which tend to show clear convergence towards maximum overlap and stable log-likelihood curves across the 20 iterations (dashed lines), as seen in **Fig 2b** in the main text, as opposed to a lack of a clear minimum and noisy incoherence across the 20 iterations. In these scenarios, we have previously concluded that MiXeR results are consistent with maximum possible overlap, although it may be possible to more precisely estimate the genetic overlap between these traits with greater statistical power (1). This is also provides a good example of the importance of visualising the log-likelihood plots while performing QC for bivariate MiXeR analyses.

Supplementary figure

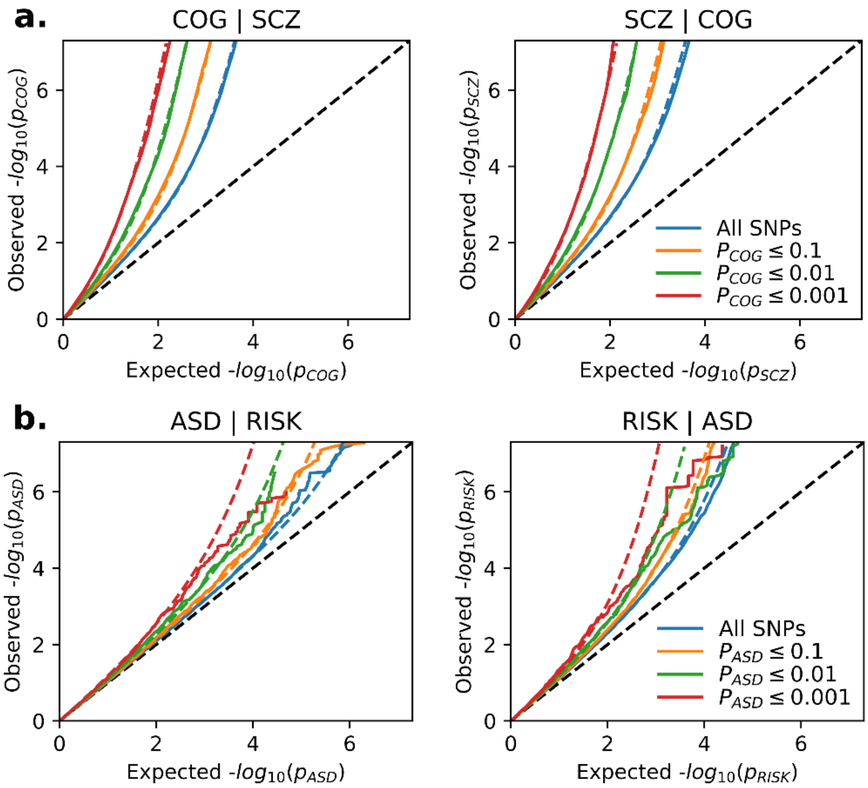

Supplementary figure 1: Stratified QQ plots comparing observed cross-trait enrichment (solid lines) to MiXeR-modelled cross-trait enrichment (dashed lines). **Panel a**. shows an example of good model fit in which the MiXeR-modelled enrichment closely maps on to the observed data. **Panel b** shows an example of poor model fit in which there is the MiXeR modelled encrichment diverges from the observed data.

Reference

1. Hindley G, Frei O, Shadrin AA, Cheng W, O’Connell KS, Icick R, *et al.* (2022): Charting the Landscape of Genetic Overlap Between Mental Disorders and Related Traits Beyond Genetic Correlation. *Am J Psychiatry* appiajp21101051.
